# Unveiling toxicological adverse outcomes: toward construction and simulation of large-scale networks

**DOI:** 10.1093/toxsci/kfag043

**Published:** 2026-04-10

**Authors:** Nensi Ikonomi, Natalie Ketter, Mario A A Pepe

**Affiliations:** Global Non-clinical Safety Science, Boehringer Ingelheim Pharma GmbH & Co. KG, Birkendorfer Strasse 65, Biberach, 88397, Germany; Global Non-clinical Safety Science, Boehringer Ingelheim Pharma GmbH & Co. KG, Birkendorfer Strasse 65, Biberach, 88397, Germany; Global Non-clinical Safety Science, Boehringer Ingelheim Pharma GmbH & Co. KG, Birkendorfer Strasse 65, Biberach, 88397, Germany

**Keywords:** large-scale network models, Boolean networks, adverse outcome prediction, molecular crosstalks, dynamic simulation

## Abstract

Predicting toxicological adverse outcomes is crucial for advancing in silico toxicology strategies. Modern toxicology increasingly relies on systems biology approaches to model and interpret these outcomes. Adverse outcome pathways (AOPs) focus on systems-level descriptions and causal linear relations among initiating, key, and adverse outcome events. Key characteristics (KC)-based topologies capture mechanistic breadth via interconnected property-based modules without assuming linear causality. From another perspective, emerging physiological maps dive deeper into toxicological mechanisms by mapping them at the detailed molecular level. To capture the dynamic nature of toxicological responses, especially their time- and dose-dependent behaviors, there is growing interest in integrating systems biology and mathematical modeling strategies. Although dynamic models have been applied to small-scale AOPs, larger regulatory networks remain largely unexplored from a dynamic perspective. In this review, we highlight recent efforts to combine systems and network biology approaches for predicting toxicological adverse outcomes, covering network construction, analysis, and dynamic predictions. We also explore the aspect of dynamically simulating large-scale molecular networks and its potential contribution to systems toxicology. Specifically, we charter the use of logic-based models (Boolean networks) as an integrative approach to understand molecular crosstalk and cellular phenotypes, highlighting the potential repurpose of existing models. To this end, we show 2 use cases on toxicological applications of Boolean network models. Finally, we prospectively discuss the importance and need of bridging molecular and systemic scales and integrating these modeling strategies with high-dimensional data sources, including omics and multi-omics datasets.

The view of living organisms as integrated systems and interrelated components has shaped the field of systems biology. This holistic paradigm includes interactions between organisms and xenobiotics, which lays the conceptual foundation for sub-areas such as systems toxicology. Systems toxicology integrates traditional toxicological approaches with quantitative analysis of extensive networks of molecular and functional alterations across various biological levels. It aims to describe the resilience of biological systems to toxicant-induced perturbations and their ability (or lack thereof) to re-establish physiological function. This intrinsic interdisciplinary nature is central to unraveling complex interactions within biological systems (Sturla et al. 2014; Hartung et al. 2017). To understand and link toxicants to adverse outcomes (AOs), it is essential to summarize and reconstruct the mechanisms leading to these events. Systems theory postulates that interactions store more information than individual elements, centralizing attention on the adoption of network-based strategies (del Giudice et al. 2024).

In this contemporary review, we aim to present an overview of recent trends in network-based approaches in systems toxicology. We examine the challenges in designing and analyzing large-scale networks, incorporating examples from current research in systems toxicology.

In the first part of this review, we provide an overview of recent trends in network creation, from small to large-scale networks, and across different biological layers. Traditionally, adverse outcome pathways (AOPs) have led to the adoption of network-oriented methods. We begin with a summary of this paradigm, which models systemic mechanisms at the tissue, organ, individual, and even population level, and then describe the expansion into larger-scale AOP networks (AOPNs). Moving from the AOP paradigm, alternative systemic topologies such as KC networks are evaluated. Beyond the systemic networks, there is an increasing demand in toxicology for a mechanistic understanding at the gene and cellular level (Staumont et al. 2025). To address this, we focus further on modeling strategies that aim to extensively map molecular interactions, such as protein–protein interactions (PPIs) and the newly emerging physiological maps.

In the second part, we detail the analysis of such networks, highlighting challenges and limitations, especially when approaching the dynamic simulation of larger-scale systems. Here, in dedicated paragraphs, we further charter the possibility of employing a yet unseen strategy of dynamic simulation in toxicology, focusing on logic-based modeling. Furthermore, we provide 2 use cases showcasing how to utilize the framework to address mechanisms of toxicity and feed back its results into systemic-level topologies. Finally, we consider future perspectives, discussing synergies among network modeling approaches, simulation strategies, and the bridging to large-scale data. The 2 parts are summarized graphically in Fig. 1.

**Fig. 1.**
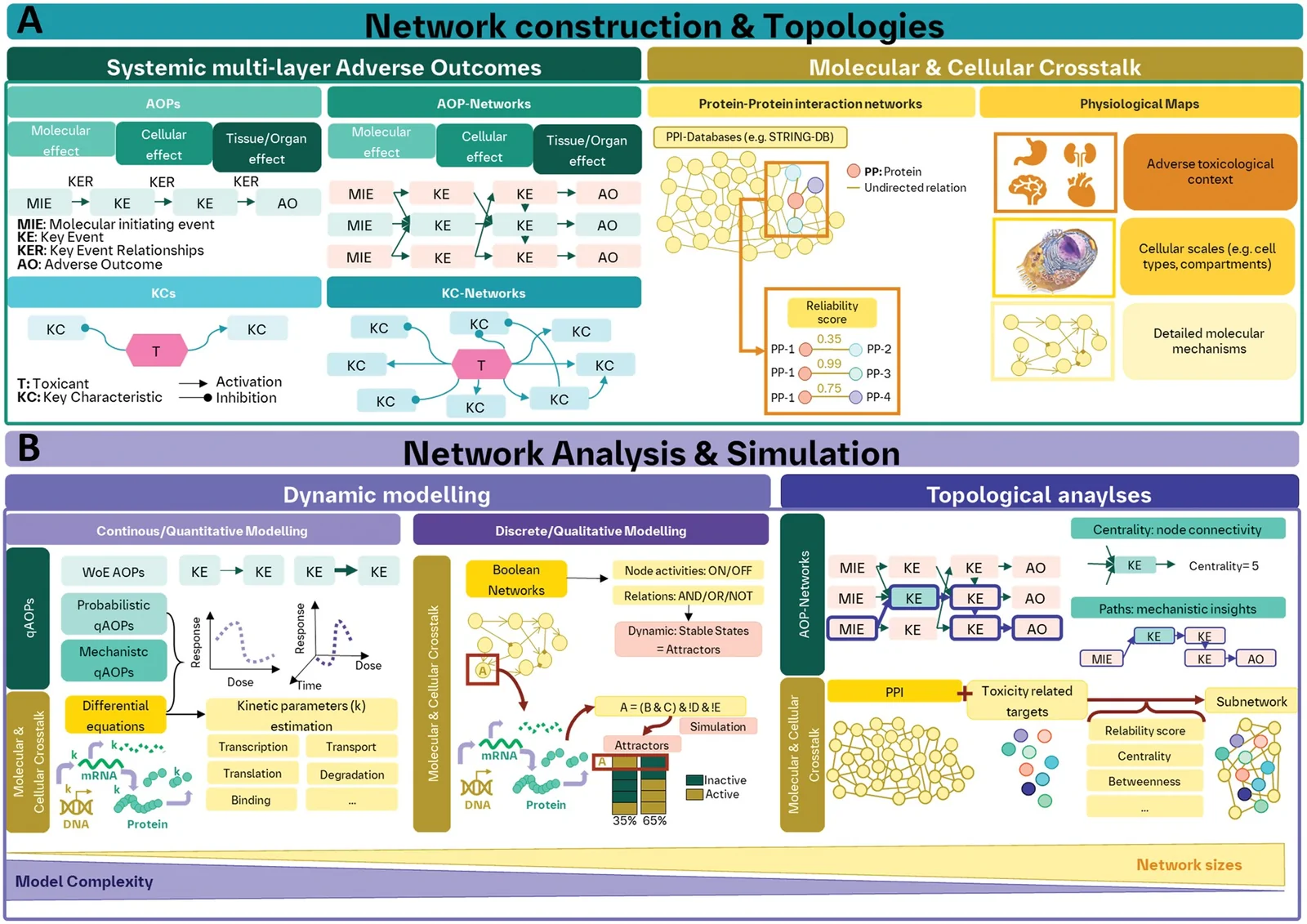
Overview of network construction and analysis strategies in systems toxicology. A) Network construction and topologies spanning systemic multi-layer adverse outcomes (left) and molecular/cellular crosstalk (right). Systemic representations include AOPs and AOPN, organizing information as interconnected MIEs, KEs, and AOs, as well as KC networks, which structure mechanistic evidence into interconnected KC modules linked to a toxicant elucidating the activation of end-points. Molecular/cellular representations include PPI networks (illustrated with database-derived interaction reliability scores) and physiological maps, which contextualize detailed molecular mechanisms across cellular scales and adverse toxicological contexts. B) Network analysis and simulation strategies ranging from dynamic modeling (left) to topological analyses (right). Dynamic approaches include continuous/quantitative modeling (e.g. qAOPs and kinetic/ODE-based descriptions) and discrete/qualitative modeling (Boolean networks), yielding stable states/attractors. Topological investigation is illustrated with examples for AOPNs and PPIs. The 2 gradients below the analysis panel indicate the inverse relationship between network size (yellow) and model/analysis complexity (purple).

## Mapping toxicity AOs: from AOPs and KCs to PPIs and physiological maps

Ankley et al. (2010) hypothesized the concept of AOPs, which has become a gold-standard network strategy for understanding toxicological mechanisms. The AOP framework pragmatically describes and organizes existing biological knowledge into directed acyclic cascades of events, from a molecular initiating event (MIE) to an AO. The cascade comprises a series of key events (KEs), causally linked by key event relationships (KERs) (Zgheib et al. 2019; Jaylet et al. 2024). Additionally, AOPs span different regulatory levels of biological organization, from a MIE to an organ or systemic effect (Zgheib et al. 2019; Jaylet et al. 2024) (Fig. 1A, left panel).

Considering the challenges in deriving such systems, significant effort has been devoted to establishing best practices and strategies for constructing AOPs (Villeneuve et al. 2014a, 2014b). Specifically, the central need for modularity and reusability of KE and KERs is recognized (Villeneuve et al. 2014a, 2014b). This aspect is particularly important because a single linear AOP likely represents an oversimplification of both the complexity of biological systems and the downstream events related to stressors. In other words, the “one perturbation, one adverse outcome” model that a linear AOP represents does not fit the real-world scenario of stress exposure and the multiplicity of related mechanisms of toxicity (Villeneuve et al. 2014a; Knapen et al. 2018). Thus, AOPNs, composed of single AOPs sharing common KEs and KERs, have become increasingly relevant as functional units for toxicity prediction (Villeneuve et al. 2014a; Knapen et al. 2018), especially after the release of the AOP-Wiki collecting published AOPs (Martens et al. 2018) (Fig. 1A, left panel). Following this principle, Pollesch et al. (2019) assembled all 187 AOPs present in the AOP-Wiki (as of April 2018) into a single network comprising 840 unique KEs and 1,050 KERs. From the fusion of the full set of AOPs, the authors tracked the emergence of 9,405 unique and previously undescribed linear AOPs. Alternatively, AOPNs can be built starting from the selection and combination of AOPs from a specific area of interest, such as biological context, MIEs, or AOs (Knapen et al. 2018). In this context, the selection and integration are manually curated and tailored a priori to a specific toxicological question. Recent examples of such tailored strategies are presented by Spinu et al. (2019) and Barnes et al. (2024), who, respectively, developed AOPNs for neurotoxicity and nephrotoxicity. Considering the rapid development of new AOPNs, the need for computational tools to support their construction, handling, and integration has increased. Recent examples of such tools include AOPExplorer, designed as a Cytoscape plugin to visualize and explore AOPNs, and AOP-helpFinder, which leverages artificial intelligence to automatically screen the available literature supporting the development of AOPs (Jornod et al. 2022; Jaylet et al. 2023). Further, Effectopedia is a knowledge aggregation and modeling platform designed for the collaborative development and utilization of AOPs (https://norecopa.no/3rset-resources/introduction-to-effectopedia).

Beyond AOPs, which structure mechanistic knowledge into directed acyclic cascades, the KCs framework provides a complementary, non-sequential approach for organizing mechanistic evidence across diverse toxicological endpoints (Smith et al. 2016, 2020; Smith 2025) (Fig. 1A, left panel). KCs describe the inherent mechanistic properties of agents known to induce AOs without presupposing a defined temporal order. Instead, they offer a property-based representation of biological perturbations that may occur independently, concurrently, or in combinations that vary across stressors. This makes KCs particularly useful where mechanistic perturbations arise from distributed or partially independent processes, such as in carcinogenesis, where DNA damage, altered proliferation, immunomodulation, and redox imbalance often operate in parallel and cannot be reliably assigned to a single linear AOP cascade (Smith et al. 2016, 2020; Smith 2025). The KC framework has been expanded beyond carcinogens to include reproductive toxicants, endocrine disrupting chemicals, hepatotoxicants, and cardiovascular toxicants (Smith et al. 2016; Guyton et al. 2018; Arzuaga et al. 2019; Luderer et al. 2019; Lind et al. 2021; Rusyn et al. 2021; Scholten et al. 2022; Merrill et al. 2025). KC-based networks are constructed by first identifying the characteristic biological properties relevant to the agent class and then mapping the molecular, cellular, and systemic evidence that supports each KC. Considering the example of benzene-induced carcinogenicity, extensive mechanistic literature links benzene metabolites to DNA damage, chromosomal aberrations, oxidative stress, bone marrow immunomodulation, metabolic dysregulation, and altered cell proliferation (Smith et al. 2016; Scholten et al. 2022). These biological properties populate KC-specific modules, whereas causal relationships extracted from experimental and epidemiological studies define the edges connecting them within the carcinogenicity network. Tools such as INDRA (Integrated Network and Dynamical Reasoning Assembler) facilitate this process by automatically mining mechanistic relations from publications and databases and assembling them into structured, computable causal graphs (Gyori et al. 2017). When applied to the benzene-induced carcinogenicity case, INDRA-derived networks already capture interactions among DNA damage responses, oxidative stress pathways, inflammatory mediators, and immune dysregulation, providing a robust starting scaffold (Scholten et al. 2022). Automated extractions are then complemented by manual curation to refine KC modules, assess evidence strength, resolve redundancies, and ensure alignment with the defined KC set. Altogether, the KC-based networks complement AOPs: although AOPs work well with evidence-based causal chains, KC-based networks capture a broader set of mechanisms, particularly when processes are distributed, partially independent, or not yet linearly resolved. Prospectively, the integration of both approaches could support the construction of mechanistic topologies spanning to systemic levels that preserve causal clarity where it is known and incorporate mechanistic breadth where complexity prevails (Smith 2025).

Although AOPs and KCs model mechanisms of toxicity at a systemic, multi-layered biological level, other network topologies are emerging and utilized for unveiling toxicity mechanisms at a molecular crosstalk level. PPI networks are large relational networks collecting information on regulatory dependencies (edges) among proteins (nodes) at a genomic scale (Fig. 1A, right panel). Recently, several authors have utilized these networks to identify molecular targets involved in toxicity. They do this by reconstructing sub-networks from human PPIs, starting from a set of proteins of interest and expanding outward (Chu and Zi 2024; Huang 2024; Lin et al. 2024; Cui et al. 2025; Li et al. 2025; Qu et al. 2025). The most utilized PPI is STRING PPIs, that currently collects almost 20,000 nodes and over 5.5 million edges for the human PPI network (Szklarczyk et al. 2023). The main advantage of employing STRING includes the possibility of accessing specific reliability scores for protein–protein relations; thus, the user can further filter and customize the generated PPI (Szklarczyk et al. 2023). Exemplarily, Qu et al. (2025) utilized the STRING PPI to reconstruct a specific molecular sub-network sourced from STRING-DB composed of 1,004 proteins and 1,839 edges (Qu et al. 2025). Mostly such PPIs are currently handled via the software Cytoskape and related specific plug-ins (Shannon et al. 2003; Otasek et al. 2019).

As an additional large-scale network, the recently presented physiological map conceptualized by Staumont et al. (2025) integrates the need for detailed molecular and cellular mechanisms with the link to specific organ physiological functions (Fig. 1A, right panel). Sourcing from the lessons learned and effort of the disease map project (Mazein et al. 2018; Ostaszewski et al. 2021), the physiological map concept not only designs the strategy for the construction of large-scale and multi-granularity-level networks but also foresees the use of graphical standardized outputs, such as the Systems Biology Graphical Notation (Novère et al. 2009) and the further plug-in into interactive user interfaces such as the MINERVA platform (Gawron et al. 2016). The construction strategy presented for the physiological maps comprises a tight interplay between domain experts and curators in the planning of the biological mechanism to be described and the further inclusion and documentation of key components as cell types, molecules, and pathways (Staumont et al. 2025).

## Modeling dynamics of AOs: simulating and analyzing small- and large-scale models

Once any network topology is established, the main interest is to analyze the resulting model, especially by designing a dynamic (quantitative) mathematical modeling strategy. In the context of linear AOPs, quantitative AOPs (qAOPs) are defined as predictive computational models that provide a dose and/or time dynamic response to toxicants (Zgheib et al. 2019; Spinu et al. 2020). qAOPs are generally categorized in 3 conceptual classes: (i) weight of evidence semi-quantitative (qWoE) AOPs, (ii) Probabilistic qAOPs, and (iii) mechanistic AOPs (Spinu et al. 2020). qWoE AOPs employ quantitative weighting and multiple lines of evidence to rank the confidence in KERs for further quantification (Spinu et al. 2020) (Fig. 1B, left panel). Probabilistic AOPs employ computational models that incorporate statistic or probabilistic approaches, such as Bayesian Networks (Spinu et al. 2020; Ito et al. 2024). Finally, mechanistic qAOP are deterministic systems where mathematical functions are used to describe relations between MIEs, KEs, and AOPs (Spinu et al. 2020). Zgheib et al. (2019) compared different qAOPs for the description of the same toxicological outcome, highlighting their advantages and limitations. Specifically, they compared dose–response modeling with a dynamic Bayesian network approach. They emphasized that both approaches require suitable experimental data for model construction, and interpretations of the models must remain within the dose ranges used for predictions. Bayesian networks represent a sophistication of the dose–response fitting, allowing them to investigate more complex behaviors. However, they also face limitations, particularly the inability to incorporate feedback loops. Given their high data requirements and reliance on linear causal relationships, the above-mentioned dynamic strategies are best suited for modeling linear AOPs. Simulation of such systems is available on platforms such as Effectopedia.

When moving to larger-scale networks, such as AOPNs and PPIs-derived ones, quantitative dynamic of time and/or dose responses become increasingly prohibitive with the increase of network sizes. For this reason, AOPN and PPI analyses mostly focus on network topological measures, such as degree centrality and path calculations, with specific declination for AOPNs (Villeneuve et al. 2018; Pollesch et al. 2019) (Fig. 1B, right panel). For the PPIs, these topological measures are mostly employed in optimizing the identification of relevant sub-networks from a set of targets of interest (Chu and Zi 2024; Huang 2024; Lin et al. 2024; Cui et al. 2025; Li et al. 2025; Qu et al. 2025). Additionally, Valls-Margarit et al. (2023) further explore a benchmark of refined network biology methods to analyze and identify such sub-networks for toxicological mechanistic investigations.

Such network analyses can be performed in Cytoskape and related specific plug-ins (Shannon et al. 2003; Otasek et al. 2019). However, the employment of mathematical modeling for the description of larger networks is yet largely unexplored for toxicological approaches, especially when investigating molecular networks and mechanisms.

## Accessing large-scale dynamics: charting the employment of Boolean networks for prediction of toxicological outcomes

As toxicological research increasingly demands mechanistic insight into complex biological responses, traditional modeling approaches often struggle to scale or generalize. This chapter explores how Boolean networks offer a practical and mechanistically informative alternative for predicting AOs.

When considering the need for larger models to mechanistically understand and predict toxicological AOs, it is beneficial to explore systems biology-based mathematical modeling solutions to represent their dynamics. Zgheib et al. (2019) describe the modeling via differential equations of a molecular network describing oxidative stress, representing the detailed kinetics of around 25 genes/molecules and their products. Although this strategy enables modeling of complex feedback loops and detailed molecular mechanisms, it is constrained by complexity and substantial data requirements for parameterization (Zgheib et al. 2019). Exemplarily, in the above-mentioned use case, the final established model counted 57 differential equations and 355 parameters, all of which required estimation (Zgheib et al. 2019). On these grounds, we chart the use of an alternative mathematical strategy for simulating large-scale dynamics, particularly when extensive kinetic data are unavailable (Bloomingdale et al. 2018; Hemedan et al. 2022) (Fig. 1B, left panel). Boolean networks are a mathematical modeling approach that has gained increasing popularity in the systems biology community (Fig. 1B, left panel). By simplifying node activities to binary ON/OFF states and regulatory dependencies as logic gates (AND/OR/NOT), Boolean networks enable simulation of large dynamic systems while retaining key dynamic outcomes of corresponding kinetic models (Lavrova et al. 2017; Paulevé et al. 2020; Schwab et al. 2020). This simplification allows model construction when only qualitative information is available, without requiring new experimental efforts. The design of such systems also allows for the representation of different semantic levels, e.g. cellular phenotypes such as apoptosis, proliferation, or senescence. The dynamic simulation of Boolean networks leads to the identification of one or more possible stable states (called attractors) that are directly related to biologically motivated phenotypes. Attractors (phenotypes) can be further quantified in terms of their probability of occurring by estimating the number of initially simulated states flowing into them (basin of attraction) (Schwab et al. 2020; Ikonomi et al. 2022). This enables comparison of phenotypes/attractors across perturbation conditions (e.g. physiological versus toxicant-induced) and quantification of their occurrence probabilities. Additionally, the study of network trajectories can support the hypothesis of dynamically driven mechanisms (Ikonomi et al. 2022). Considering the recent advancements in execution paradigm Boolean networks simulations, the dynamic exploration of attractors has scaled up to potentially genomic-scale network sizes (Paulevé et al. 2020). The use of Boolean network as a modeling strategy has already been described from a systems pharmacology perspective (Bloomingdale et al. 2018; Hemedan et al. 2022). In this domain, specific tools have been already developed to enrich the dynamic description of node relations beyond ON/OFF and extended perturbation strategies that could also support their application for toxicological purposes (Irurzun-Arana et al. 2017). Beyond this, the flexibility of the Boolean network framework allows for the creation of hybrid models that can incorporate continuous data when available, supported by established simulation platforms (Stoll et al. 2017). As an employment strategy for toxicological purposes, Boolean networks can be used in tandem to physiological maps efforts as a simulation strategy, similarly to the disease map projects (Ostaszewski et al. 2021; Mazein et al. 2023).

## On the practical use of Boolean networks in toxicology: framework and use cases

Although Boolean models remain largely unexplored in toxicology, many existing models already capture pathways and phenotypes relevant to toxicant responses. Collaborative repositories such as Cell Collective (Helikar et al. 2012), which hosts ∼90 curated models, facilitate the identification and reuse of suitable models for toxicological questions. Here, we outline a practical workflow for repurposing Boolean models to interrogate toxicological outcomes and present 2 use cases: One related to toxicant exposure-induced carcinogenesis and one addressing drug-induced adverse events.

**Fig. 2.**
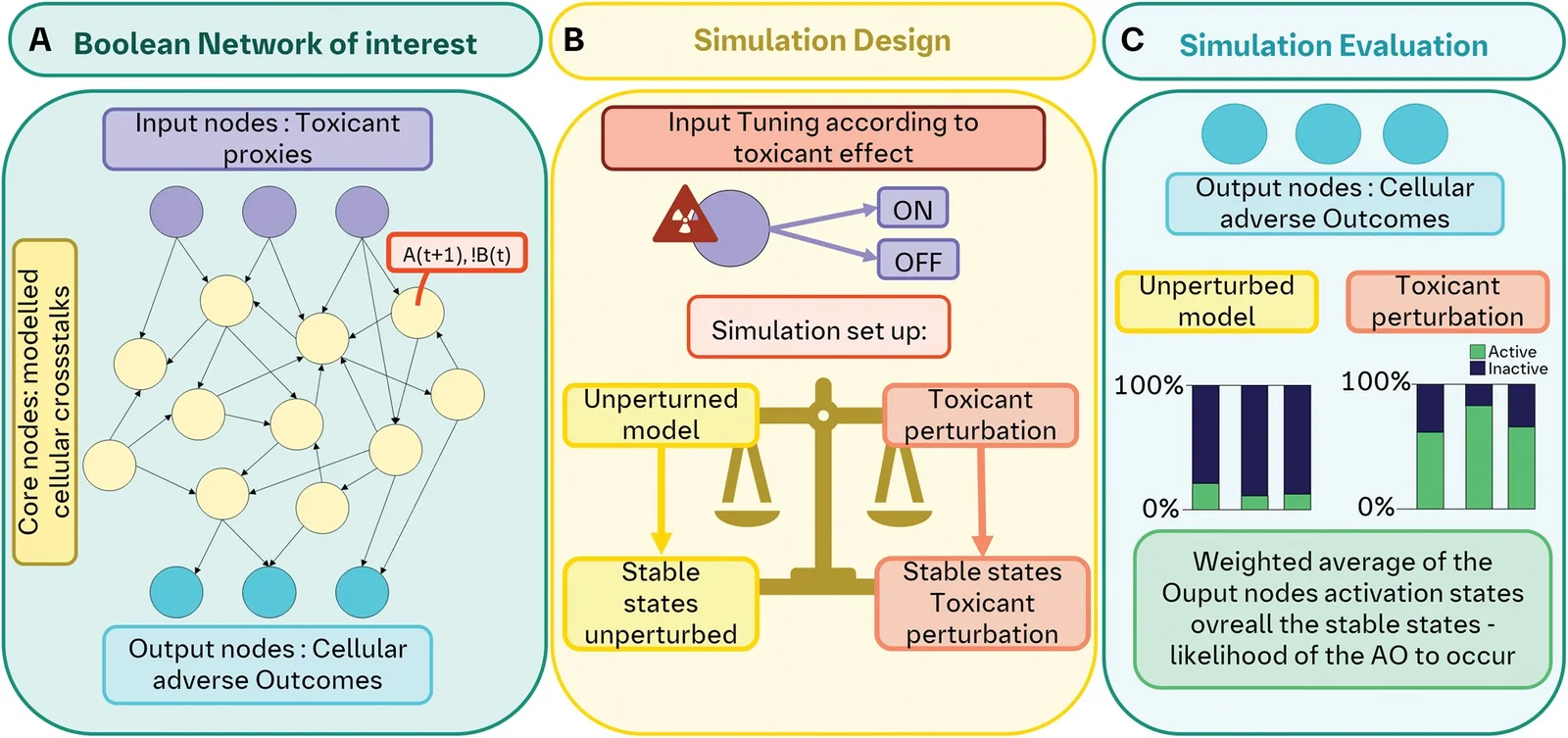
Boolean network workflow for evaluating toxicological relevant perturbations. A) Schematic graph representation of a Boolean network illustrating input nodes corresponding to toxicant proxies, core regulatory nodes capturing cellular crosstalk, and output nodes representing cellular adverse outcomes. B) Simulation design in which toxicant-related perturbations are introduced at input nodes and stable states are computed for both unperturbed and perturbed settings. C) Evaluation of simulation results by comparing activation states of output nodes across attractors, providing an estimated likelihood of adverse cellular outcomes under toxicant perturbation.

When repurposing a Boolean model, the first step is to select a model that reflects the pathways and cellular outcomes relevant to the toxicological question (Fig.  2A). A crucial step in this phase is the identification of an appropriate entry point for introducing a toxicant proxy. In straightforward cases, this may correspond to an input node; however, any node in the network can serve as a perturbation target if biologically justified. For drug-related AOs, the perturbed node is typically the drug’s molecular target or a downstream component known to be activated or inhibited by the drug. In the context of environmental or carcinogenic exposures, suitable entry points may include nodes representing DNA damage or reactive oxygen species. After this, comparative simulations are conducted, unperturbed (physiological-like) versus toxicant-like perturbations, to observe changes in steady states (Fig.  2B). Paired simulations (unperturbed vs. toxicant perturbation) are run. The results are then compared through basins of attraction for output phenotypes of interest (Fig. 2C). Differences in basins abundance directly indicate whether a perturbation increases or decreases the likelihood of a given cellular outcome, such as apoptosis, proliferation, migration, or senescence. For example, one can evaluate whether a drug-like perturbation raises or lowers the probability of apoptosis relative to the physiological (unperturbed) state. These simulations provide a mechanistic foundation to guide targeted follow-up studies, refine AOP hypotheses, and strengthen early hazard identification. By revealing how defined perturbations shift cellular phenotypes, they help identify mechanistic liabilities, compare alternative mechanisms, and prioritize experimental work based on predicted phenotypic shifts.

The first use case focuses on toxicant-induced carcinogenicity. Considering the KC-based carcinogenicity network developed by Scholten et al. (2022), each KC can be represented as a functional module, and the relationships between KCs can be formalized as logical or Boolean rules, enabling simulation of signaling convergence and emergent toxicodynamic states. This translation can be supported directly by INDRA, which derives Boolean functions from the mechanistic statements used to assemble the network. Such a model assembly would generate a high-level dynamic representation of the KC-based network. Alternatively, it is possible to use each KC with its relation as a dynamic unit: The KC network provides a structured chain of mechanistic relations that can be directly mapped to a modular set of Boolean networks. For example, the authors report that “*benzene activates DNA damage* and that *DNA damage activates cell death*, *while cell death activates inflammatory responses*” and “*disruption of the cell cycle can lead to apoptotic processes*”. Each of these mechanistic units corresponds to well-studied input–output relationships (e.g. “DNA damage” as input and “apoptosis” or “cell-cycle arrest” as outputs) for which standalone Boolean models already exist (Calzone et al. 2010; Lin et al. 2014; Sizek et al. 2019; Gupta et al. 2024). By selecting logic models that capture these biological transitions, it is possible to “walk” through benzene’s toxicological mode of action: Each model (or set of models) feeds into the next one using the KC relationships to set the simulation conditions.

As a second use case, drug-related AOs were explored using the hepatocyte apoptosis model of Schlatter et al. (2009), applied here in the context of drug-induced liver injury (DILI). The model already encodes key stress inputs relevant to hepatotoxicity, including cytokine/death-receptor signaling, inflammatory milieu, mitochondrial/oxidative injury, and RIP-dependent signaling. Published simulations show that mild activation of these pathways leads to caspase-dependent apoptosis, whereas stronger mitochondrial damage or RIP-skewed signaling shifts the system toward necrotic outcomes, capturing dose-dependent transitions characteristic of DILI. Because these stress pathways are explicitly represented, drugs interfering with them can be mapped onto the corresponding input nodes or suitable proxies, allowing the model to simulate how they perturb the system. In this way, the use case illustrates how Boolean models can support exploring mechanistic hypotheses and predicting hepatotoxicity-related outcomes. Simulation results can be mapped into systemic networks: The cholestasis AOPN of van Ertvelde et al. (2023) serves as an illustrative example. Apoptosis and necrosis inform hepatocyte injury/death KEs, the cytokine context supports inflammation KEs, and mitochondrial/oxidative stress informs oxidative-stress KEs. Thus, the model outputs populate relevant KEs, and the AOPN functions as an integrative scaffold that translates cellular-scale logic outcomes into a systemic mechanistic progression relevant for DILI.

Together, these use cases show that Boolean network simulations provide a practical, mechanistically transparent way to evaluate toxicological hypotheses, test perturbations, anticipate adverse phenotypes, and link insights directly to AOP or KC frameworks, complementing and strengthening modern mechanistic toxicology.

## Conclusions

Our contemporary review highlights recent efforts in mechanistic modeling of systemic and molecular networks unraveling toxicological AOs. We emphasize the increasing need to capture larger topologies to better understand toxicological mechanisms. As these networks expand, current analytic approaches, largely limited to static analyses, can no longer capture the dynamic behaviors essential for mechanistic investigations. To overcome this limitation, we propose Boolean networks as a scalable qualitative modeling strategy, supported by their successful use in systems pharmacology and disease map projects and aligned with emerging physiological maps that bridge molecular and systemic levels. Through 2 use cases covering toxicant exposure and drug-induced toxicity, we demonstrate how repurposing published Boolean models enables mechanistic explorations across distinct toxicological contexts. Finally, we showcase how outputs from these dynamic models can be reintegrated into the systemic topologies of AOP- and KC-based networks, supporting a unified framework for predicting AOs.

## Future directions

Advancing systems toxicology will require deeper integration across biological scales. Although AOPs remain indispensable for structuring systemic outcomes, they should increasingly be linked to the mechanistic depth provided by KC-based networks and the molecular resolution of PPIs and physiological maps. Recent efforts to map AOP KEs directly to genes and gene sets through natural-language-processing techniques (Saarimäki et al. 2023a; Jaylet et al. 2024) exemplify this shift and open the door to incorporating multi-omics datasets (Guan et al. 2022; Perkins et al. 2022; Saarimäki et al. 2023b) into these frameworks. Platforms such as MINERVA further enable integration of high-throughput data into physiological maps (Staumont et al. 2025). In parallel, Boolean networks offer a dynamic layer that can interface with these expanding mechanistic topologies. Omics data will play an increasingly central role not only in validating Boolean models but also in deriving them, particularly through single-cell transcriptomic information (Schwab et al. 2021; Maier et al. 2024). Together, these developments point toward a multiscale modeling paradigm where molecular and cellular dynamics can be systematically aligned with AOP- and KC-based systemic structures. The convergence of large-scale mechanistic maps, omics-driven model refinement, and qualitative dynamic modeling provides a promising path toward more predictive and mechanistically grounded assessments of AOs.

## References

[kfag043-B1] Ankley GT , Bennett RS, Erickson RJ, Hoff DJ, Hornung MW, Johnson RD, Mount DR, Nichols JW, Russom CL, Schmieder PK, et al 2010. Adverse outcome pathways: a conceptual framework to support ecotoxicology research and risk assessment. Environ Toxicol Chem. 29:730–741. 10.1002/etc.34. 20821501

[kfag043-B2] Arzuaga X , Smith MT, Gibbons CF, Skakkebæk NE, Yost EE, Beverly BEJ, Hotchkiss AK, Hauser R, Pagani RL, Schrader SM, et al 2019. Proposed key characteristics of male reproductive toxicants as an approach for organizing and evaluating mechanistic evidence in human health hazard assessments. Environ Health Perspect. 127:065001. 10.1289/ehp5045. PMC6792367 31199676

[kfag043-B3] Barnes DA , Firman JW, Belfield SJ, Cronin MTD, Vinken M, Janssen MJ, Masereeuw R. 2024. Development of an adverse outcome pathway network for nephrotoxicity. Arch Toxicol. 98:929–942. 10.1007/s00204-023-03637-7. PMC10861692 38197913

[kfag043-B4] Bloomingdale P , Nguyen VA, Niu J, Mager DE. 2018. Boolean network modeling in systems pharmacology. J Pharmacokinet Pharmacodyn. 45:159–180. 10.1007/s10928-017-9567-4. PMC6531050 29307099

[kfag043-B5] Calzone L , Tournier L, Fourquet S, Thieffry D, Zhivotovsky B, Barillot E, Zinovyev A. 2010. Mathematical modelling of cell-fate decision in response to death receptor engagement. PLoS Comput Biol. 6:e1000702. 10.1371/journal.pcbi.1000702. PMC2832675 20221256

[kfag043-B6] Chu Z-Y , Zi X-J. 2024. Network toxicology and molecular docking for the toxicity analysis of food contaminants: a case of aflatoxin B1. Food Chem Toxicol. 188:114687. 10.1016/j.fct.2024.114687. 38663764

[kfag043-B7] Cui Z , He T, Zhang S. 2025. The efficient prediction of inflammatory osteolysis caused by polylactic acid through network toxicology and molecular docking strategy. Ecotoxicol Environ Saf. 291:117876. 10.1016/j.ecoenv.2025.117876. 39947065

[kfag043-B8] del Giudice G , Serra A, Pavel A, Maia MT, Saarimäki LA, Fratello M, Federico A, Alenius H, Fadeel B, Greco D. 2024. A network toxicology approach for mechanistic modelling of nanomaterial hazard and adverse outcomes. Adv Sci. 11:2400389. 10.1002/advs.202400389. PMC11348149 38923832

[kfag043-B9] Gawron P , Ostaszewski M, Satagopam V, Gebel S, Mazein A, Kuzma M, Zorzan S, McGee F, Otjacques B, Balling R, et al 2016. MINERVA—a platform for visualization and curation of molecular interaction networks. NPJ Syst Biol Appl. 2:16020. 10.1038/npjsba.2016.20. PMC5516855 28725475

[kfag043-B10] Guan R , Li N, Wang W, Liu W, Li X, Zhao C. 2022. The adverse outcome pathway (AOP) of estrogen interference effect induced by triphenyl phosphate (TPP): integrated multi-omics and molecular dynamics approaches. Ecotoxicol Environ Saf. 234:113387. 10.1016/j.ecoenv.2022.113387. 35272188

[kfag043-B11] Gupta S , Silveira DA, Lorenzoni PR, Mombach JCM, Hashimoto RF. 2024. LncRNA PTENP1/miR-21/PTEN axis modulates EMT and drug resistance in cancer: dynamic Boolean modeling for cell fates in DNA damage response. Int J Mol Sci. 25:8264. 10.3390/ijms25158264. PMC11311614 39125832

[kfag043-B12] Guyton KZ , Rieswijk L, Wang A, Chiu WA, Smith MT. 2018. Key characteristics approach to carcinogenic hazard identification. Chem Res Toxicol. 31:1290–1292. 10.1021/acs.chemrestox.8b00321. PMC6424613 30521319

[kfag043-B13] Gyori BM , Bachman JA, Subramanian K, Muhlich JL, Galescu L, Sorger PK. 2017. From word models to executable models of signaling networks using automated assembly. Mol Syst Biol. 13:954. 10.15252/msb.20177651. PMC5731347 29175850

[kfag043-B14] Hartung T , FitzGerald RE, Jennings P, Mirams GR, Peitsch MC, Rostami-Hodjegan A, Shah I, Wilks MF, Sturla SJ. 2017. Systems toxicology: real world applications and opportunities. Chem Res Toxicol. 30:870–882. 10.1021/acs.chemrestox.7b00003. PMC5396025 28362102

[kfag043-B15] Helikar T , Kowal B, McClenathan S, Bruckner M, Rowley T, Madrahimov A, Wicks B, Shrestha M, Limbu K, Rogers JA. 2012. The cell collective: toward an open and collaborative approach to systems biology. BMC Syst Biol. 6:96. 10.1186/1752-0509-6-96. PMC3443426 22871178

[kfag043-B16] Hemedan AA , Niarakis A, Schneider R, Ostaszewski M. 2022. Boolean modelling as a logic-based dynamic approach in systems medicine. Comput Struct Biotechnol J. 20:3161–3172. 10.1016/j.csbj.2022.06.035. PMC9234349 35782730

[kfag043-B17] Huang S. 2024. A novel strategy for the study on molecular mechanism of prostate injury induced by 4,4′‐sulfonyldiphenol based on network toxicology analysis. J Appl Toxicol. 44:28–40. 10.1002/jat.4506. 37340727

[kfag043-B18] Ikonomi N , Werle SD, Schwab JD, Kestler HA. 2022. TGF-beta signaling, methods and protocols. Methods Mol Biol. 2488:159–181. 10.1007/978-1-0716-2277-3_12. 35347689

[kfag043-B19] Irurzun-Arana I , Pastor JM, Trocóniz IF, Gómez-Mantilla JD. 2017. Advanced boolean modeling of biological networks applied to systems pharmacology. Bioinformatics. 33:1040–1048. 10.1093/bioinformatics/btw747. 28073755

[kfag043-B20] Ito S , Mukherjee S, Erami K, Muratani S, Mori A, Ichikawa S, White W, Yoshino K, Fallacara D. 2024. Proof of concept for quantitative adverse outcome pathway modeling of chronic toxicity in repeated exposure. Sci Rep. 14:4741. 10.1038/s41598-024-55220-4. PMC10899215 38413641

[kfag043-B21] Jaylet T , Coustillet T, Jornod F, Margaritte-Jeannin P, Audouze K. 2023. AOP-helpFinder 2.0: integration of an event-event searches module. Environ Int. 177:108017. 10.1016/j.envint.2023.108017. 37295163

[kfag043-B22] Jaylet T , Coustillet T, Smith NM, Viviani B, Lindeman B, Vergauwen L, Myhre O, Yarar N, Gostner JM, Monfort-Lanzas P, et al 2024. Comprehensive mapping of the AOP-Wiki database: identifying biological and disease gaps. Front Toxicol. 6:1285768. 10.3389/ftox.2024.1285768. PMC10958381 38523647

[kfag043-B23] Jornod F , Jaylet T, Blaha L, Sarigiannis D, Tamisier L, Audouze K. 2022. AOP-helpFinder webserver: a tool for comprehensive analysis of the literature to support adverse outcome pathways development. Bioinformatics. 38:1173–1175. 10.1093/bioinformatics/btab750. PMC8796376 34718414

[kfag043-B24] Knapen D , Angrish MM, Fortin MC, Katsiadaki I, Leonard M, Margiotta-Casaluci L, Munn S, O’Brien JM, Pollesch N, Smith LC, et al 2018. Adverse outcome pathway networks I: development and applications. Environ Toxicol Chem. 37:1723–1733. 10.1002/etc.4125. PMC6004608 29488651

[kfag043-B25] Lavrova AI , Postnikov EB, Zyubin A, Babak SV. 2017. Ordinary differential equations and Boolean networks in application to modelling of 6-mercaptopurine metabolism. R Soc Open Sci. 4:160872. 10.1098/rsos.160872. PMC5414245 28484608

[kfag043-B26] Li H , Yu B, Yuan Y, Chen N, Guo H, Zhang H, Zhang Z. 2025. Integrated computational analysis of molecular mechanisms underlying perfluorooctane sulfonic acid induced thyroid toxicity. Sci Rep. 15:7920. 10.1038/s41598-025-92678-2. PMC11885520 40050647

[kfag043-B27] Lin G-Q , Ao B, Chen J-W, Wang W-X, Di Z-R. 2014. Modeling and controlling the two-phase dynamics of the p53 network: a Boolean network approach. New J Phys. 16:125010. 10.1088/1367-2630/16/12/125010.

[kfag043-B28] Lin Z , Li Y, Zhao J, Li J, Pan S, Wang X, Lin H, Lin Z. 2024. Exploring the environmental contamination toxicity and potential carcinogenic pathways of perfluorinated and polyfluoroalkyl substances (PFAS): an integrated network toxicology and molecular docking strategy. Heliyon. 10:e37003. 10.1016/j.heliyon.2024.e37003. PMC11402918 39286118

[kfag043-B29] Lind L , Araujo JA, Barchowsky A, Belcher S, Berridge BR, Chiamvimonvat N, Chiu WA, Cogliano VJ, Elmore S, Farraj AK, et al 2021. Key characteristics of cardiovascular toxicants. Environ Health Perspect. 129:095001. 10.1289/ehp9321. PMC8462506 34558968

[kfag043-B30] Luderer U , Eskenazi B, Hauser R, Korach KS, McHale CM, Moran F, Rieswijk L, Solomon G, Udagawa O, Zhang L, et al 2019. Proposed key characteristics of female reproductive toxicants as an approach for organizing and evaluating mechanistic data in hazard assessment. Environ Health Perspect. 127:075001. 10.1289/ehp4971. PMC6791466 31322437

[kfag043-B31] Maier J , Schwab JD, Werle SD, Marienfeld R, Möller P, Gaisa NT, Ikonomi N, Kestler HA. 2024. Boolean network modeling and its integration with experimental read-outs. Die Pathol. 45:26–30. 10.1007/s00292-024-01395-6. 39535613

[kfag043-B32] Martens M , Verbruggen T, Nymark P, Grafström R, Burgoon LD, Aladjov H, Andón FT, Evelo CT, Willighagen EL. 2018. Introducing WikiPathways as a data-source to support adverse outcome pathways for regulatory risk assessment of chemicals and nanomaterials. Front Genet. 9:661. 10.3389/fgene.2018.00661. PMC6308971 30622555

[kfag043-B33] Mazein A , Acencio ML, Balaur I, Rougny A, Welter D, Niarakis A, Ardila DR, Dogrusoz U, Gawron P, Satagopam V, et al 2023. A guide for developing comprehensive systems biology maps of disease mechanisms: planning, construction and maintenance. Front Bioinform. 3:1197310. 10.3389/fbinf.2023.1197310. PMC10325725 37426048

[kfag043-B34] Mazein A , Ostaszewski M, Kuperstein I, Watterson S, Novère NL, Lefaudeux D, Meulder BD, Pellet J, Balaur I, Saqi M, et al 2018. Systems medicine disease maps: community-driven comprehensive representation of disease mechanisms. NPJ Syst Biol Appl. 4:21. 10.1038/s41540-018-0059-y. PMC5984630 29872544

[kfag043-B35] Merrill MAL , Smith MT, McHale CM, Heindel JJ, Atlas E, Cave MC, Collier D, Guyton KZ, Koliwad S, Nadal A, et al 2025. Consensus on the key characteristics of metabolism disruptors. Nat Rev Endocrinol. 21:245–261. 10.1038/s41574-024-01059-8. PMC11916920 39613954

[kfag043-B36] Novère NL , Hucka M, Mi H, Moodie S, Schreiber F, Sorokin A, Demir E, Wegner K, Aladjem MI, Wimalaratne SM, et al 2009. The systems biology graphical notation. Nat Biotechnol. 27:735–741. 10.1038/nbt.1558. 19668183

[kfag043-B37] Ostaszewski M , Niarakis A, Mazein A, Kuperstein I, Phair R, Orta‐Resendiz A, Singh V, Aghamiri SS, Acencio ML, Glaab E et al; COVID-19 Disease Map Community. 2021. COVID19 Disease Map, a computational knowledge repository of virus–host interaction mechanisms. Mol Syst Biol. 17:e10851. 10.15252/msb.202110387. PMC8696085 34939300

[kfag043-B38] Otasek D , Morris JH, Bouças J, Pico AR, Demchak B. 2019. Cytoscape automation: empowering workflow-based network analysis. Genome Biol. 20:185. 10.1186/s13059-019-1758-4. PMC6717989 31477170

[kfag043-B39] Paulevé L , Kolčák J, Chatain T, Haar S. 2020. Reconciling qualitative, abstract, and scalable modeling of biological networks. Nat Commun. 11:4256. 10.1038/s41467-020-18112-5. PMC7450094 32848126

[kfag043-B40] Perkins EJ , Woolard EA, Garcia-Reyero N. 2022. Integration of adverse outcome pathways, causal networks and ‘omics to support chemical hazard assessment. Front Toxicol. 4:786057. 10.3389/ftox.2022.786057. PMC8987526 35399296

[kfag043-B41] Pollesch NL , Villeneuve DL, O’Brien JM. 2019. Extracting and benchmarking emerging adverse outcome pathway knowledge. Toxicol Sci. 168:349–364. 10.1093/toxsci/kfz006. PMC10545168 30715536

[kfag043-B42] Qu T , Sun Q, Tan B, Wei H, Qiu X, Xu X, Gao H, Zhang S. 2025. Integration of network toxicology and transcriptomics reveals the novel neurotoxic mechanisms of 2, 2′, 4, 4′-tetrabromodiphenyl ether. J Hazard Mater. 486:136999. 10.1016/j.jhazmat.2024.136999. 39740552

[kfag043-B43] Rusyn I , Arzuaga X, Cattley RC, Corton JC, Ferguson SS, Godoy P, Guyton KZ, Kaplowitz N, Khetani SR, Roberts RA, et al 2021. Key characteristics of human hepatotoxicants as a basis for identification and characterization of the causes of liver toxicity. Hepatology. 74:3486–3496. 10.1002/hep.31999. PMC8901129 34105804

[kfag043-B44] Saarimäki LA , Fratello M, Pavel A, Korpilähde S, Leppänen J, Serra A, Greco D. 2023a. A curated gene and biological system annotation of adverse outcome pathways related to human health. Sci Data. 10:409. 10.1038/s41597-023-02321-w. PMC10290716 37355733

[kfag043-B45] Saarimäki LA , Morikka J, Pavel A, Korpilähde S, Giudice G, del Federico A, Fratello M, Serra A, Greco D. 2023b. Toxicogenomics data for chemical safety assessment and development of new approach methodologies: an adverse outcome pathway‐based approach. Adv Sci (Weinh). 10:e2203984. 10.1002/advs.202203984. PMC9839874 36479815

[kfag043-B46] Schlatter R , Schmich K, Vizcarra IA, Scheurich P, Sauter T, Borner C, Ederer M, Merfort I, Sawodny O. 2009. On/off and beyond—a Boolean model of apoptosis. PLoS Comput Biol. 5:e1000595. 10.1371/journal.pcbi.1000595. PMC2781112 20011108

[kfag043-B47] Scholten B , Simón LG, Krishnan S, Vermeulen R, Pronk A, Gyori BM, Bachman JA, Vlaanderen J, Stierum R. 2022. Automated network assembly of mechanistic literature for informed evidence identification to support cancer risk assessment. Environ Health Perspect. 130:037002. 10.1289/ehp9112. PMC8893280 35238605

[kfag043-B48] Schwab JD , Ikonomi N, Werle SD, Weidner FM, Geiger H, Kestler HA. 2021. Reconstructing Boolean network ensembles from single-cell data for unraveling dynamics in the aging of human hematopoietic stem cells. Comput Struct Biotechnol J. 19:5321–5332. 10.1016/j.csbj.2021.09.012. PMC8487005 34630946

[kfag043-B49] Schwab JD , Kühlwein SD, Ikonomi N, Kühl M, Kestler HA. 2020. Concepts in Boolean network modeling: what do they all mean? Comput Struct Biotechnol J. 18:571–582. 10.1016/j.csbj.2020.03.001. PMC7096748 32257043

[kfag043-B50] Shannon P , Markiel A, Ozier O, Baliga NS, Wang JT, Ramage D, Amin N, Schwikowski B, Ideker T. 2003. Cytoscape: a software environment for integrated models of biomolecular interaction networks. Genome Res. 13:2498–2504. 10.1101/gr.1239303 PMC403769 14597658

[kfag043-B51] Sizek H , Hamel A, Deritei D, Campbell S, Regan ER. 2019. Boolean model of growth signaling, cell cycle and apoptosis predicts the molecular mechanism of aberrant cell cycle progression driven by hyperactive PI3K. PLoS Comput Biol. 15:e1006402. 10.1371/journal.pcbi.1006402 PMC6436762 30875364

[kfag043-B52] Smith MT. 2025. The key characteristics concept. Curr Opin Toxicol. 41:100515. 10.1016/j.cotox.2024.100515. PMC12017793 40270588

[kfag043-B53] Smith MT , Guyton KZ, Gibbons CF, Fritz JM, Portier CJ, Rusyn I, DeMarini DM, Caldwell JC, Kavlock RJ, Lambert PF, et al 2016. Key characteristics of carcinogens as a basis for organizing data on mechanisms of carcinogenesis. Environ Health Perspect. 124:713–721. 10.1289/ehp.1509912. PMC4892922 26600562

[kfag043-B54] Smith MT , Guyton KZ, Kleinstreuer N, Borrel A, Cardenas A, Chiu WA, Felsher DW, Gibbons CF, Goodson WH, Houck KA, et al 2020. The key characteristics of carcinogens: relationship to the hallmarks of cancer, relevant biomarkers, and assays to measure them. Cancer Epidemiology Prev Biomark. 29:1887–1903. 10.1158/1055-9965.epi-19-1346. PMC7483401 32152214

[kfag043-B55] Spinu N , Bal-Price A, Cronin MTD, Enoch SJ, Madden JC, Worth AP. 2019. Development and analysis of an adverse outcome pathway network for human neurotoxicity. Arch Toxicol. 93:2759–2772. 10.1007/s00204-019-02551-1. 31444508

[kfag043-B56] Spinu N , Cronin MTD, Enoch SJ, Madden JC, Worth AP. 2020. Quantitative adverse outcome pathway (qAOP) models for toxicity prediction. Arch Toxicol. 94:1497–1510. 10.1007/s00204-020-02774-7. PMC7261727 32424443

[kfag043-B57] Staumont B , Ladeira L, Gamba A, Heusinkveld HJ, Piersma A, Fritsche E, Masereeuw R, Vanhaecke T, Teunis M, Luechtefeld TH, et al 2025. Mapping physiology: a systems biology approach for the development of alternative methods in toxicology. ALTEX. 42:301–307. 10.14573/altex.2412241. 39918919

[kfag043-B58] Stoll G , Caron B, Viara E, Dugourd A, Zinovyev A, Naldi A, Kroemer G, Barillot E, Calzone L. 2017. MaBoSS 2.0: an environment for stochastic Boolean modeling. Bioinformatics. 33:2226–2228. 10.1093/bioinformatics/btx123. 28881959

[kfag043-B59] Sturla SJ , Boobis AR, FitzGerald RE, Hoeng J, Kavlock RJ, Schirmer K, Whelan M, Wilks MF, Peitsch MC. 2014. Systems toxicology: from basic research to risk assessment. Chem Res Toxicol. 27:314–329. 10.1021/tx400410s. PMC3964730 24446777

[kfag043-B60] Szklarczyk D , Kirsch R, Koutrouli M, Nastou K, Mehryary F, Hachilif R, Gable AL, Fang T, Doncheva NT, Pyysalo S, et al 2023. The STRING database in 2023: protein–protein association networks and functional enrichment analyses for any sequenced genome of interest. Nucleic Acids Res. 51:D638–D646. 10.1093/nar/gkac1000. PMC9825434 36370105

[kfag043-B61] Valls-Margarit J , Piñero J, Füzi B, Cerisier N, Taboureau O, Furlong LI. 2023. Assessing network-based methods in the context of system toxicology. Front Pharmacol. 14:1225697. 10.3389/fphar.2023.1225697. PMC10369070 37502213

[kfag043-B62] van Ertvelde J , Verhoeven A, Maerten A, Cooreman A, Rodrigues BdS, Sanz-Serrano J, Mihajlovic M, Tripodi I, Teunis M, Jover R, et al 2023. Optimization of an adverse outcome pathway network on chemical-induced cholestasis using an artificial intelligence-assisted data collection and confidence level quantification approach. J Biomed Inform. 145:104465. 10.1016/j.jbi.2023.104465. 37541407

[kfag043-B63] Villeneuve DL , Angrish MM, Fortin MC, Katsiadaki I, Leonard M, Margiotta-Casaluci L, Munn S, O’Brien JM, Pollesch NL, Smith LC, et al 2018. Adverse outcome pathway networks II: network analytics. Environ Toxicol Chem. 37:1734–1748. 10.1002/etc.4124. PMC6010347 29492998

[kfag043-B64] Villeneuve DL , Crump D, Garcia-Reyero N, Hecker M, Hutchinson TH, LaLone CA, Landesmann B, Lettieri T, Munn S, Nepelska M, et al 2014a. Adverse outcome pathway (AOP) development I: strategies and principles. Toxicol Sci. 142:312–320. 10.1093/toxsci/kfu199. PMC4318923 25466378

[kfag043-B65] Villeneuve DL , Crump D, Garcia-Reyero N, Hecker M, Hutchinson TH, LaLone CA, Landesmann B, Lettieri T, Munn S, Nepelska M, et al 2014b. Adverse outcome pathway development II: best practices. Toxicol Sci. 142:321–330. 10.1093/toxsci/kfu200. PMC4318924 25466379

[kfag043-B66] Zgheib E , Gao W, Limonciel A, Aladjov H, Yang H, Tebby C, Gayraud G, Jennings P, Sachana M, Beltman JB, et al 2019. Application of three approaches for quantitative AOP development to renal toxicity. Comput Toxicol. 11:1–13. 10.1016/j.comtox.2019.02.001.

